# Fast Analysis of Caffeic Acid-Related Molecules in Instant Coffee by Reusable Sonogel–Carbon Electrodes

**DOI:** 10.3390/s22218448

**Published:** 2022-11-03

**Authors:** Laura Pigani, Cristina Rioli, Barbara Zanfrognini, Juan José García-Guzmán, José Maria Palacios-Santander, Laura María Cubillana-Aguilera

**Affiliations:** 1 Dipartimento di Scienze Chimiche e Geologiche, Università degli Studi di Modena e Reggio Emilia, Via G. Campi, 103, 41125 Modena, Italy; 2 Instituto de Investigación e Innovación Biomédica de Cadiz (INiBICA), Hospital Universitario ‘Puerta del Mar’, Universidad de Cadiz, 11009 Cadiz, Spain; 3 Institute of Research on Electron Microscopy and Materials (IMEYMAT), Department of Analytical Chemistry, Faculty of Sciences, Campus de Excelencia Internacional del Mar, University of Cadiz, Campus Universitario de Puerto Real, Polígono del Río San Pedro S/N, 11510 Puerto Real, Cadiz, Spain

**Keywords:** electrochemical sensor, Sonogel–Carbon electrode, carbon black, coffee, caffeic acid, polyphenols, differential pulse voltammetry

## Abstract

Reusable Sonogel–Carbon electrodes containing carbon black (SNGC-CB) have been used for the electrochemical analysis of caffeic acid (CA) in real matrices. Measurements were firstly performed in standard solutions, in which SNGC-CB electrodes allowed the electrochemical determination of CA with high sensitivity and low limit of detection, equal to 0.76 μM. The presence of CB nanostructures in the formulation led to improved performances with respect to pristine SNGC electrodes. Then, measurements were performed in four instant coffees of different brands. A comparison between the results obtained by electrochemical, chromatographic and spectroscopic methods showed that SBGC-CB electrodes represent a simple and economic tool for the rapid assessment of caffeic acid-related molecules in instant coffees.

## 1. Introduction

Polyphenols are a common constituent of foodstuffs and beverages, mainly of vegetal origin, affecting quality, stability, acceptability, aging as aromatizing, colorants and antioxidants [1,2]. The presence of two or more phenolic groups allows them to act as reducing agents, donating protons or electrons to radical species, thus lowering their toxic properties [1,3]. The polyphenolic content in foodstuffs of vegetable origin is extremely variable, from 1 mg/L in white wine and beers to 100 mg/L in red wines, fruit juices, coffees, teas and cacao, as a function of the food typologies, production methods and geographical origin [4].

The two main groups of polyphenolic compounds are phenolic acids and flavonoids. Phenolic acids are hydroxylated derivatives of benzoic and cinnamic acids, and they exist either in free form or preferably bound as glycosides. Chlorogenic acids are the most prominent polyphenolic compounds in coffee drinks. Chemically, they are a family of different esters formed between trans-cinnamic acid, among which are caffeic acid (CA) and quinic acid [5]. The most abundant chlorogenic acid in coffee is 5-O-caffeoylquinic acid, often indicated simply as CGA. CA itself, thanks to the presence of a catechol group in its molecule, is one of the most effective polyphenolic antioxidants helping to prevent some diseases caused by oxidative stress [6].

In recent decades, the food industry has required the development of a high number of analytical methods for the identification and quantification of polyphenols in foodstuffs. The determination of the single phenolic compounds has been investigated by using various analytical methods, such as high-performance liquid chromatography [7], liquid chromatography–mass spectrometry [8] and capillary zone electrophoresis [9]. At the same time, the determination of the total polyphenolic content (TPC) can be very useful in order to define the value of a food due to the beneficial effects associated with the presence of polyphenols. The most common procedure used for the determination of TPC is represented by the Folin–Ciocalteu method, based on the reaction between the polyphenols and a colorimetric reagent able to induce absorbance increase in the visible region of the spectrum [10].

Electrochemical methods have recently emerged as simple and fast alternatives for the determination of both specific polyphenolic substances and antioxidant properties of foodstuffs. The electrochemical index (EI) has been introduced to define the total phenolic content determined by electrochemistry [11,12,13]. Due to the electroactive nature of antioxidant polyphenols, they can be easily electrochemically oxidized while forming appropriate quinoid compounds. Electrochemical techniques are characterized by high performance in terms of the limit of detection and sensitivity and the possibility of operating in complex, semisolid and turbid samples without the addition of reagents and/or expensive instrumentation [14]. The literature reports numerous examples of amperometric sensors developed for the determination of the antioxidant properties of foodstuffs in terms of CA or CGA content [15,16,17,18,19,20]. However, one of the most important limitations in the electrochemical determination of polyphenols is given by the fouling of the electrode surface.

In a recent paper, we demonstrated the efficacy of Sonogel–Carbon electrodes containing carbon black (SNGC-CB electrodes) in the electrochemical detection of small polyphenolic molecules, such as catechol [21]. The presence of CB, a carbonaceous nanostructured material, induces electrocatalytic phenomena, which are particularly evident with catechol-like molecules [22]. The main characteristic of the SNGC electrodes lies in the possibility of adding different components to the graphite phase to obtain a material characterized by peculiar electrocatalytic properties. In this way, the active component is homogeneously dispersed in the bulk electrode material and not only located at the surface. As a first advantageous consequence, in the occurrence of passivation phenomena, the electrode surface can be easily renewed by a simple scratching leading to reproducible results [23,24]. The SNGC electrodes can then be used for a high number of measurements, with a view to saving and reusability. In our recent publication regarding SNGC-CB electrodes, we also demonstrated that thanks to an efficient electrochemical cleaning procedure in KOH solution, fouling phenomena typical when dealing with polyphenolic species can be avoided, assuring high repeatability and reproducibility. The same electrode can indeed be used several times for the analysis of many different samples [21]. On the basis of these results, in this paper, we verified the possibility of testing SNGC-CB electrodes in the investigation of other polyphenolic molecules found in real samples, such as instant coffee. Coffee is one of the most consumed beverages in the world and is the major source of dietary CA intake in humans [5]. The content of CA and CGA is strictly connected to several parameters, among which the variety of coffee beans, the roasting conditions and the preparation methods of the coffee beverages [25,26] are relevant. Here, we propose a simple and rapid electrochemical procedure for the determination of one of the major markers of coffee samples, CA, paying particular attention to the matrix effect.

## 2. Materials and Methods

### 2.1. Reagents and Materials

All chemicals were of reagent grade. Ultra-pure deionized Milli-Q water (18 MΩ cm resistivity, Millipore, Bedford, MA, USA) was used for the preparation of all solutions.

For the preparation of SNGC-CB electrodes methyltrimethoxysilane (Merck, Darmstadt, Germany), HCl (Merck), graphite powder (natural, high purity, 200 Mesh, 99.999% metal basis, Alfa Aesar, Karlsruhe, Germany) and CB N220 (Cabot Corporation, Ravenna, Italy) were used.

NaOH (98%, Fisher Scientific, Loughborough, UK), monobasic and dibasic potassium phosphate salts (Carlo Erba, Cornaredo, Italy), acetic acid (99.8%, Carlo Erba) and tartaric acid (99.5%, Aldrich) were used to prepare aqueous phosphate buffer solutions (0.1 M PB, pH = 7.4), tartrate buffer solutions (0.1 M TB, pH = 3.0) and acetate buffer solutions (0.1 M AB, pH = 4.0 and 0.1 M AB, pH = 5.0), respectively.

### 2.2. Electrode Preparation Procedure

SNGC-CB electrodes were prepared according to the procedure described in ref. [21]. Briefly, a mixture of 300 mg of graphite /150 mg of CB was added to a sonosol obtained after sonicating 0.5 mL methyltrimethoxysilane and 0.1 mL of 0.2 M HCl for 5 s. The resulting material was then inserted and compacted inside a glass capillary tube, i.d. 1.15 mm, and dried before use. Electrical contact was established by inserting a copper wire in the glass capillary tube. The surface of the electrodes was polished first with emery paper (1200 mesh, Struers, Germany), then with fine paper to obtain a smooth surface and finally thoroughly washed with deionized water. The geometric area of the resulting electrodes was 1.04 × 10^−2^ cm^2^. Before use, electrodes were activated by performing 5 cyclic voltammetric scans in the potential range (−0.50–1.50) V in a 0.05 M H_2_SO_4_ solution (50 mV/s potential scan rate).

### 2.3. Apparatus and Procedures

All electrochemical measurements were performed with an Autolab PGSTAT 12 potentiostat (Ecochemie, Utrecht, The Netherlands). Voltammetric experiments were carried out in a single-compartment three-electrode cell composed of an SNGC-CB working electrode, an aqueous Ag/AgCl/3M KCl reference electrode (Metrohm, Herisau, Switzerland) and a glassy carbon rod auxiliary electrode. All measurements were performed at room temperature, under an Ar atmosphere, in order to minimize the presence of interfering oxygen in the solution. Cyclic voltammetry (CV) and differential pulse voltammetry (DPV) were used for the electrochemical tests. The DPV waveform consisted of 10 mV potential impulse, 4 mV potential step, 0.15 s pulse time, and 0.6 s time interval between two subsequent potential pulses.

After each measurement in CA and coffee solutions, an electrochemical polishing procedure was necessary in order to restore the electrode surface. SNGC-CB electrodes underwent 6 CV scans in a 17 mM KOH, 0.1 M LiClO_4_ aqueous solution, pH = 12 (50 mV/s scan rate). The cleaning procedure used between the various measurements proved to be very efficient, leading to an excellent RSD% value equal to 0.4%, indicating very high repeatability. No mechanical cleaning of the electrode surface was necessary between measurements in different solutions.

TPC was determined by the classical Folin–Ciocalteu method, using the relevant reagent (Carlo Erba); the absorbance of the phosphotungstic–phosphomolybdenum complex formed by the reaction, was measured by a Lambda750 UV-Vis spectrophotometer (Perkin Elmer, Waltham, MA, USA) at the wavelength of 750 nm.

Finally, high-performance liquid chromatography (HPLC) was performed by a Hewlett Packard series 1050 chromatograph coupled to diode-array detection (Palo Alto, CA, USA); a reverse-phase X Select C18 column (Waters, Milford, MA, USA) was used for the separation.

### 2.4. Sample Preparation

Four commercial instant coffees were considered in this study. Coffee #1, coffee #2 and coffee #3 were instant coffees of different brands; coffee #4 was a decaffeinated coffee of the same brand as coffee #2.

The coffee extracts were prepared by infusion of ca. 2 g of each sample in 25 mL of hot deionized water for 10 min under stirring. Finally, the resulting solutions were then properly diluted in 0.1 M AB, pH = 4.

## 3. Results and Discussion

### 3.1. Determination of CA at SNGC-CB Electrodes

To investigate the analytical performance of the SNGC-CB electrode as a sensing platform, we firstly detected CA in synthetic samples by means of CV and DPV. As mentioned in the Introduction section, CA is an analyte of interest due to its importance in health and agri-food fields; therefore, the development of fast and cheap analytical devices for its quantification is of paramount importance.

**Scheme 1:**
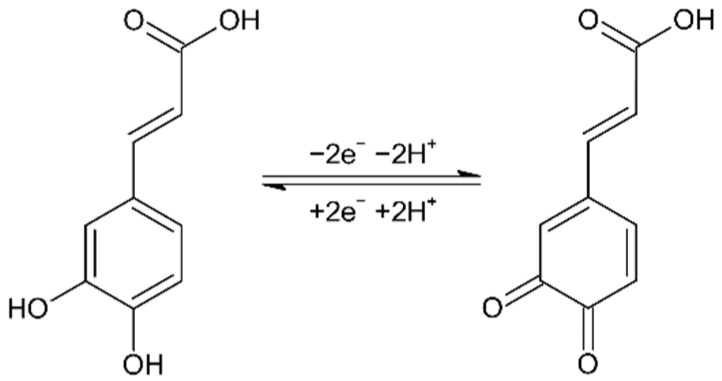
Electrochemical oxidation mechanism of CA.

Scheme 1 reports the two electrons oxidation mechanism and double proton transfer occurring during CA oxidation. Then, the pH of the solution plays an important role in its electrochemical detection.

**Figure 1:**
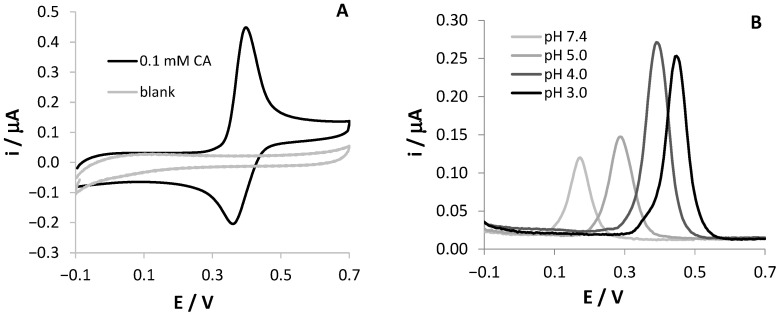
Voltammetric signals of 0.1 mM CA, registered at SNGC-CB electrodes: (**A**) CV scan in 0.1 M AB, pH = 4.0 solution; (**B**) DPV scans in solutions at different pH values ranging from 3.0 to 7.4.

Figure 1A shows the CV response of an SNGC-CB electrode in a 0.1 mM CA, 0.1 M AB, pH = 4.0 solution; the CV scan recorded in the blank solution is reported in the same graph. As expected, the voltammetric signal indicates a reversible redox process for CA. The effect of the solution pH on the electrochemical reaction at the SNGC-CB electrode was investigated by recording DPV scans at four different pH values between 3.0 and 7.4.

As shown in Figure 1B, the peak potentials were dependent on the solution pH and shifted negatively with the increase in pH. A linear regression equation of Ep (V) = –0.063 pH + 0.636, with a correlation coefficient of 0.996, describes the relationship between the anodic peak potential and the solution pH value. The slope close to the theoretical value of –59 mV demonstrates that the number of electrons and protons taking part in the electrode reaction is equal, confirming literature results [16,18]. This finding can be related to a Nernstian behavior of CA at the modified electrode. Since the oxidation of CA involves two electrons, the number of protons is two as well. On the other hand, the highest peak current value was obtained at pH 4.0, which was chosen for further experiments in order to reach higher sensitivity. At this pH, the electrochemical oxidation process of CA at the SNGC-CB electrode proved to be diffusive, as proved by the slope of the log i_p_ vs. log v being equal to 0.54 (figures not shown).

**Figure 2:**
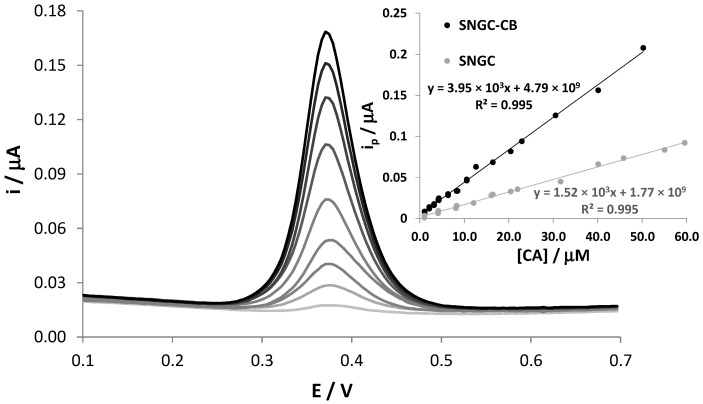
DPV signals of SNGC-CB electrode in 0.1 M AB pH = 4.0 solution in the presence of different concentrations of CA. Inset: calibration curves obtained with SNGC and SNGC-CB electrodes.

The application of SNGC-CB electrodes to the determination of CA was further investigated by DPV measurements at different concentrations of the analyte in the range of 0.1 µM to 0.1 mM, showing a linear trend in the range 1–50 µM. Figure 2 shows that the electrochemical peak current, located at +0.38 V, increases with the concentration values of CA. The graph of the peak current versus CA concentration is reported as inset. In order to evidence the electrocatalytic properties of CB, the calibration line obtained using unmodified SNGC electrodes, used as a reference, is reported in the same graph. It is evident that the presence of CB induces electrocatalytic effects, leading to much higher current values recorded during the oxidation of CA than those recorded with unmodified SNGC electrodes. Similar effects were also observed in our previous work concerning catechol and hydroquinone [21]. The linear regression equation related to the variation of the anodic peak current with CA concentration when using SNGC-CB electrodes was as follows:

I (A) = (3.95 ± 0.11) × 10^−3^ [CA] + (4.8 ± 1.9) × 10^−9^ (1)

with a correlation coefficient of R^2^ = 0.995_3_ (S_y/x_ = 3.45 × 10^−9^, 26 points overall, 95% confidence level). The sensitivity evaluated from the slope of the calibration plot of the SNGC-CB sensor was 0.380 A × M^−1^ cm^−2^, much higher than that relative to the SNGC sensor, equal to 0.147 A × M^−1^ cm^−2^. The limit of detection (LOD) was 0.76 × 10^−6^ M and was assessed as follows: 3.3 × SD_a_/b, where b and SD_a_ are the slope and estimated standard deviation of the intercept of the regression line, respectively. It is important to note that the calibration curve was obtained by analyzing solutions with different concentrations in random order. After each measurement, corresponding to a single point in the regression line, the SNGC-CB electrode underwent electrochemical cleaning in a 17 mM KOH, 0.1 M LiClO_4_ aqueous solution, pH = 12. The linear distribution of the points evidences once again the efficiency of the cleaning procedure adopted. Moreover, some data were collected using the other two different SNGC-CB electrodes; some replicated measurements correspond to almost perfectly overlapped points in the graph, scarcely distinguishable. The high reproducibility of the measurements also indicates a very high reproducibility in the procedure used for the preparation of the electrodes.

The results shown in Table 1 underline that the figures of merit of the analytical performance of the SNGC-CB electrodes are similar or better in some cases when compared with those reported in the literature. In particular, the simplicity of the preparation procedure of the SNGC-CB electrode and the possibility to easily renew the electrode surface constitutes an important added value with respect to other sensors having more complicated surface modification architectures.

### 3.2. Real Samples Analysis

**Figure 3:**
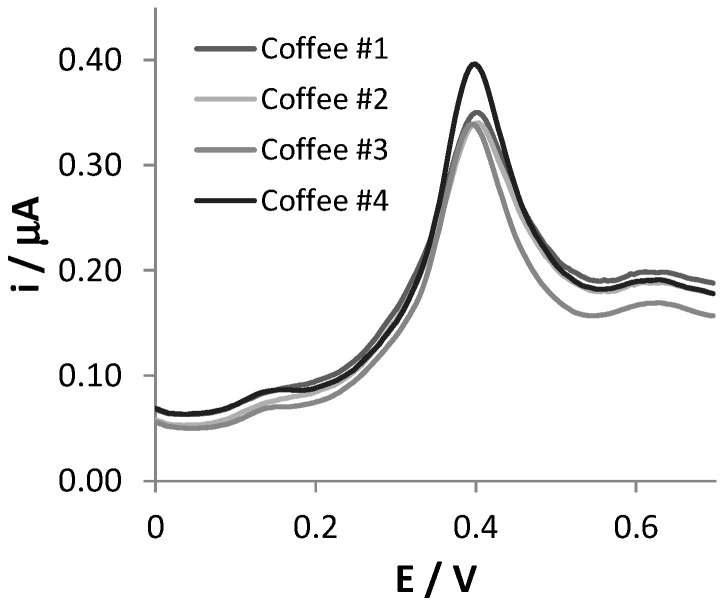
DPV scans recorded at SNGC-CB electrodes in coffee samples, diluted 1:16 in 0.1 M AB pH = 4.0 solution.

In order to assess the analytical applicability of the SNGC-CB sensor, four different commercial samples of soluble coffee powders were analyzed. Solutions of the coffee samples were prepared as described in the Materials and Methods section and properly diluted in 0.1 AB, pH = 4.0. All quantitative detections were performed in 1:1000 diluted coffee samples, but in order to better evidence the features of the DPV signals, the voltammetric scans recorded in 1:16 diluted coffee samples are reported in Figure 3.

The DPV responses showed a limited number of peaks and shoulders, making their interpretation simple. The voltammetric scans of the four typologies of coffees are very similar regarding both the position of the peaks and their current intensities. For each sample, a main peak centered at +0.41 V is well visible in the voltammetric trace, and the potential is compatible with that of CA electrooxidation. The two less evident shoulders, located at +0.17 V and +0.63 V in the voltammograms, can be ascribed to the oxidation of other redox active substances present in coffee samples, such as feruloylquinic acid, as reported in the literature [29].

**Figure 4:**
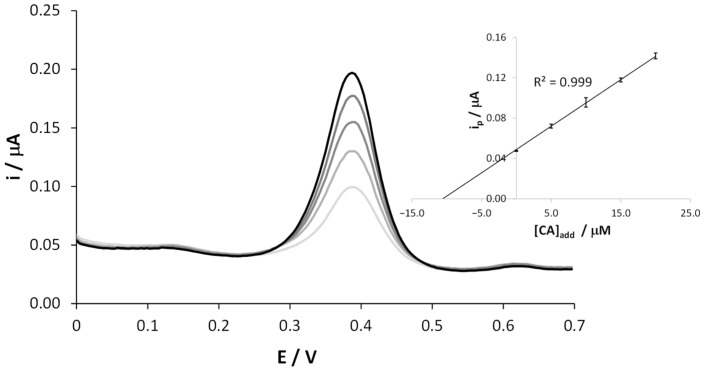
Representative DPV scans recorded at SNGC-CB electrodes in a coffee sample, diluted 1:1000 in 0.1 M AB pH = 4.0 solution, as it is and after 4 subsequent standard additions of CA. Inset: calibration curve.

The CA content in the coffee samples was determined by means of the standard addition method and the average of duplicate measurements. Thus, several aliquots of CA were added to the 1:1000 diluted coffee solution, and DPVs were recorded in the potential range between 0.0 and +0.7 V. Figure 4 reports a typical plot in which the anodic oxidation peak current increased linearly with the added amounts of CA. A calibration plot obtained considering data obtained by two distinct replicates of the same coffee brand is depicted in the inset of Figure 4. The extremely narrow error bars in the regression line account for the high reproducibility of the results. For this set of measurements, as in the previous case, no mechanical cleaning step of the electrode surface was performed, but only an electrochemical one was necessary.

The increase of the peak current at +0.4 V after each addition of CA is well visible, while the other two shoulders did not change, confirming their attribution to different polyphenolic species.

Similar plots were obtained by performing replicated measurements of the same coffee and with the other coffee samples. The results, expressed as the average of duplicate measurements, are reported in Table 2. The standard addition method is commonly applied to assess the presence of the matrix effect, which usually affects the slope of the calibration curve. Interestingly, in this case, the slopes are very similar (coffe#1: 4.57 × 10^−3^ A M^−1^; coffe#2: 4.41 × 10^−3^ A M^−1^; coffe#3: 4.48 × 10^−3^ A M^−1^; coffe#4: 4.66 × 10^−3^ A M^−1^) suggesting a similar composition of the different samples. Table 2 also reports the CA computed on the basis of the external calibration curve (Equation (1). It is evident that very similar results are obtained, demonstrating that the coffee matrix has a low impact on the CA detection using SNGC-CB electrodes.

This fact also suggests the possibility of employing the external calibration curve for rapid estimation of the CA content in coffee samples, notably reducing the analysis time since the same calibration curve can be used for the analysis of different samples. Furthermore, the recovery factors R%, computed with respect to the CA content evaluated with the standard addition method, are really satisfactory, as shown in Table 2.

In order to verify the reliability of the electrochemical method, the same coffee samples were analyzed by spectroscopic and chromatographic methods. The results obtained with the three methods are reported in Table 3. A calibration curve for HPLC methods was built using CA as a standard. For the spectroscopic measurement, the Folin–Ciocalteu method was employed, and total polyphenolic content was expressed in CA units.

As evident in Table 3, the electrochemical, spectroscopic and chromatographic techniques gave very different results. The TPC content of the four coffee samples was very similar and in agreement with the values reported in the literature for instant coffees, equal to 146–151 mg/g [4]. It is well known that the Folin–Ciocalteu method is aspecific and gives an indication of the overall content of the polyphenolic substances, so it is not surprising that electrochemical data are much lower than the TPC contents estimated by spectroscopic methods. As regards HPLC estimations of CA, these are in accordance with scientific reports as well, since in general, CA in coffee ranges between 0.21 and 2.42 mg/g [25] and measurements performed specifically in instant coffees reveal the presence of CA at a concentration of 0.12 mg/g [20]. The results obtained by the electrochemical method can be explained, considering that the overall class of chlorogenic acid compounds is detected by the SNGC-CB electrode. Actually, CGAs and CA are among the most important polyphenolic substances inside coffee, and their electrochemical signals recorded at SNGC-CB electrodes are almost identical in terms of peak potential position and current intensity (figure not shown). CGAs can usually range between 20 and 40 mg/g in instant coffees [20,29,30], in good accordance with our results. Moreover, the higher electrochemical value was found in coffe#4, the decaffeinated sample: this result is consistent with literature findings, revealing that the decaffeination process produces an increase in the total level of CGAs in coffees [31]. The experimental results confirm that a very significant contribution to the redox behavior of coffee beverages is governed by “catechol-like” major markers, which are recognized by their great antioxidant properties.

## 4. Conclusions

From these studies, it was concluded that SNGC-CB electrodes can be efficiently used for the analysis of complex matrices, such as coffee. The same electrode can be used several times, and fouling effects can be avoided by a simple cleaning procedure in a basic solution. The electrodes tested in this study have been employed for a couple of months, maintaining the high repeatability of the responses. Under the conditions developed, a simple dilution of the sample is sufficient to perform the electrochemical analysis, and the possibility of utilizing an external calibration curve for the quantification reduces to a few minutes the overall duration of the analysis.

The comparison between electrochemical, chromatographic and spectroscopic techniques affirms that the developed sensor is able to determine a specific class of polyphenolic substances, which is among the most meaningful ones in coffee, in relation to the antioxidant capacity of the beverage. 

## Figures and Tables

**Table 1:** Comparison of the analytical performances of the SNGC-CB sensor with those of other electrochemical sensors reported in the literature.

Sensor Type ^1^	Linear Range (μM)	LOD (μM)	Refs.
Ag-PEDOT/GCE	2–100	1.9	[16]
PCMTP/GCE	0.01–10	0.00534	[27]
SPE-CB	1–50	0.8	[22]
PGE	0.10–3000	0.0883	[6]
MoS_2_/GA/GCE	0.001–10.0	0.3	[19]
WS_2_/catechin-capped AuNPs/CB	0.3–112.0	0.10	[28]
SNGC	1–50	1.8	This work
SNGC-CB	1–50	0.76	This work

^1^ PEDOT = poly(3,4-ethylenedioxythiophene), PCMTP = porous carbon materials derived from *Tetrapanax papyriferus*, GCE = glassy carbon electrode, SPE = screen-printed electrode, PGE = pencil graphite electrode, GA = graphene aerogel, WS_2_/catechin-capped AuNPs/CB = tungsten disulfide flakes decorated with catechin-capped gold nanoparticles carbon black based sensor.

**Table 2:** Concentration values of CA expressed as CA/coffee mg/g computed by external calibration and by standard addition method.

Coffee	CA/Coffee (mg/g) (External Calibration)	CA/Coffee (mg/g) (Standard Addition)	R%
#1	23.2 ± 0.5	23.4 ± 0.1	99.0
#2	24.2 ± 1.3	23.6 ± 1.5	102.7
#3	24.4 ± 0.4	24.2 ± 1.7	100.8
#4	35.4 ± 1.2	33.14 ± 1.7	107.5

**Table 3:** Comparison between the results of different analytical techniques.

Coffee	DPV (External Calibration) CA/Coffee (mg/g)	HPLC CA/Coffee (mg/g)	Folin–Ciocalteu TPC as CA/Coffee (mg/g)
#1	23.2	0.21	161
#2	24.2	0.21	160
#3	24.4	0.23	166
#4	35.4	0.26	158

## Data Availability

Data is contained within the article.
